# Theory of reactive interventions in the elimination and control of malaria

**DOI:** 10.1186/s12936-019-2882-z

**Published:** 2019-08-02

**Authors:** Nakul Chitnis, Peter Pemberton-Ross, Josh Yukich, Busiku Hamainza, John Miller, Theresa Reiker, Thomas P. Eisele, Thomas A. Smith

**Affiliations:** 1 Department of Epidemiology and Public Health, Swiss Tropical and Public Health Institute, 4051 Basel, Switzerland; 2 University of Basel, Petersplatz 1, Basel, Switzerland; 3 Present Address: Amgen Europe GmbH: Rotkreuz, Zug, Switzerland; 4 Center for Applied Malaria Research and Evaluation, Tulane University, School of Public Health and Tropical Medicine, New Orleans, LA USA; 5 National Malaria Control Centre, Ministry of Health, Lusaka, Zambia; 6 PATH Malaria Control and Evaluation Partnership in Africa (MACEPA), Lusaka, Zambia

**Keywords:** Reactive case detection, Mathematical modelling, Elimination

## Abstract

**Background:**

Reactive case detection (RCD) is an integral part of many malaria control and elimination programmes and can be conceived of as a way of gradually decreasing transmission. However, it is unclear under what circumstances RCD may have a substantial impact on prevalence, how likely it is to lead to local elimination, or how effective it needs to be to prevent reintroduction after transmission has been interrupted.

**Methods:**

Analyses and simulations of a discrete time compartmental susceptible-infectious-susceptible (SIS) model were used to understand the mechanisms of how RCD changes transmission dynamics and estimate the impact of RCD programmes in a range of settings with varying patterns of transmission potential and programme characteristics. Prevalence survey data from recent studies in Zambia were used to capture the effects of spatial clustering of patent infections.

**Results:**

RCD proved most effective at low prevalence. Increasing the number of index cases followed was more important than increasing the number of neighbours tested per index case. Elimination was achieved only in simulations of situations with very low transmission intensity and following many index cases. However, RCD appears to be helpful in maintaining the disease-free state after achieving malaria elimination (through other interventions).

**Conclusion:**

RCD alone can eliminate malaria in only a very limited range of settings, where transmission potential is very low, and improving the coverage of RCD has little effect on this range. In other settings, it is likely to reduce disease burden. RCD may also help maintain the disease-free state in the face of imported infections. Prevalence survey data can be used to estimate a targeting ratio (the ratio of prevalence found through RCD to that in the general population) which is an important determinant of the effect of RCD.

**Electronic supplementary material:**

The online version of this article (10.1186/s12936-019-2882-z) contains supplementary material, which is available to authorized users.

## Background

The effects of different interventions against malaria have different temporal dynamics. Spectacular short-term effects do not necessarily result in elimination or in long-term transmission reduction, but both preventive (such as insecticide-treated nets, indoor residual spraying, intermittent preventive treatment) and curative (mass treatment) interventions deployed at fixed coverage generally have their maximal impact on the reproduction number at the start of the programme. This applies even when deployment is recurrent [1], unless operations improve over time. Irrespective of the initial level of transmission, the chances of interrupting transmission with such approaches are consequently maximized by concentrating resources in a pulse of intervention or front-loading the programme. This was recommended practice in the mid-twentieth century [2, 3].

In contrast to this, the recent history of malaria programmes seems to indicate that elimination requires sustained intervention over long periods [4]. In some cases, this may reflect a cognitive bias: if malaria disappeared because of environmental change associated with gradual socio-economic development (one plausible explanation for ‘stickiness’ of elimination [5]), then it would be mistaken to attribute success to the long-term maintenance of a programme of preventive interventions alongside development in other sectors. A well-timed short-lived programme (or even business as usual) might have been more efficient.

An alternative or complementary explanation for long timelines is that intervention programmes have had impacts on the reproduction number that increased over time. One way in which this may have occurred is that impacts may cumulate with interventions with long durations. For instance, distribution of long-lasting insecticidal nets (LLINs) at a rate greater than their attrition rate, or larval source reduction by incrementally removing breeding sites may lead to gradual increases in impact, eventually leading to environments that cannot sustain transmission.

Another way of gradually decreasing transmission, leading to a steady advance towards elimination may be reactive intervention deployment. Approaches are reactive if there is targeting in space or time in response to information gained in the course of the programme. Targeting and containment of local epidemics was the main approach of the only successful eradication programme for a human infection to date, that of smallpox [6], and it is tempting to argue by analogy that a similar strategy should be used for malaria. However, in many areas, malaria typically has a very high basic reproduction number and often has a large asymptomatic reservoir, which means that the logistical challenge of finding infections is much greater than for smallpox. Malaria is also an endemic infection for which both preventive and curative interventions are possible, as opposed to an epidemic disease against which the only interventions were preventive, so there is a wider range of possible reactive strategies against malaria. The reactions might range from treatment of passively detected cases, to follow-up of cases with test, treat and/or focal vector control of household members and/or their neighbours, to targeted mass drug administration. The most obvious reactive intervention is to treat people living near to the home of a clinical case (with or without diagnostic testing). This is often called reactive case detection (RCD).

Effective surveillance-response (i.e. reactive) strategies are essential in final stages of an elimination programme, since persistence of the disease-free-state depends both on detecting imported infections and preventing their spread [7]. If elimination is achieved in the absence of such capacity it will be impossible to verify that transmission is interrupted, and undetected reintroductions will be inevitable. This is implicitly understood by the managers of successful programmes, such as those of Morocco [8] or Sri Lanka [9]. However, theoretical analyses of elimination strategies have neglected the possibility that reactive interventions may also have been an essential part of the package that reduced transmission to levels where this endgame was possible.

This paper considers the theory of reactive intervention strategies applied in malaria intervention programmes with the analysis of a simple discrete-time susceptible-infectious-susceptible (SIS) model [10] of infection that does not consider heterogeneity in transmission potential in time or between hosts. The analysis here considers the asymptotic dynamics of this model and the qualitative changes to the asymptotic dynamics resulting from reactive strategies to better understand the role that these strategies have on elimination. The paper introduces three different models for reactive interventions, culminating with one parameterized with data from Southern Zambia [11, 12]. Although there is a long history of dynamical models of malaria transmission [13–15], there has been little focus to date on such reactive interventions. A notable exception, using a spatially explicit stochastic individual-based simulation model suggested that case management was more effective than RCD in low transmission settings; and vector control and case management should be the focus in higher transmission settings, with RCD or mass treatment used to reduce the asymptomatic reservoir [16].

The distinguishing characteristic of reactive interventions is that they are deployed selectively in time and space, in response to surveillance data. This selectivity means that the number of infections that are addressed at any one time-point is inflated above the number that would be addressed if the intervention was applied indiscriminately. In the models proposed here for RCD, this effect is captured by inflating the number of infections treated with a quantity termed the targeting ratio, defined as the ratio between the number of infected individuals treated by the reactive component, to the number that would have been treated had the selection been a simple random sample rather than neighbours of the index cases. The targeting ratio is thus approximately equivalent to the ratio of the prevalence of infection in the neighbourhood of an index case to the prevalence in the general population. The targeting ratio is a single parameter that quantifies the clustering of malaria infections due to spatial heterogeneity. Using the Zambian data, it is shown how this quantity can be estimated from survey data obtainable before the RCD is initiated, making it feasible to predict the dynamic effect of an RCD programme from cross-sectional data obtainable in advance without the need for detailed spatial analysis techniques (which are often difficult for programmes to conduct).

## Methods

### SIS model of transmission dynamics

The state variable, parameters and notation of the SIS model are given in Table 1.

**Table 1:** Model parameters and notation

Symbol	Parameter	Assumed value or limits	Source
$I_{t}$	Number of infectious individuals at time *t*	$0 \le I_{t} \le 10000$	
$N$	Total human population	$N = 10,000$	Approximate population of Zambian health centre catchment
$\beta$	Transmission parameter, i.e. the potential number of new infections per infected individual at the next time step	$\beta > 0$	
$\gamma$	Proportion of infectious population that remains infectious at next time step (which includes the removal of infections through passive detection)	$0 < \gamma < 1$	
$\varphi_{t}$	Number of infections detected (and treated) at time *t*	$\varphi_{t} > 0$	
$\varepsilon_{t}$	Escape probability: the proportion of infections that escape treatment at time *t*	$0 < \varepsilon_{t} < 1$	
$p$	Proportion of population that is (patently) infected	$0 < p < 1$	
$\upiota$	Number of index cases investigated per unit time	$\upiota > 0$	
$\upnu$	Number of neighbours of passively-detected index cases investigated	$0 < \nu < N$	Fixed property of programme
$\uptau$	Targeting ratio: ratio of the size of a random sample that would be need to be tested and treated, to the number actually treated, in order to achieve the same number of effective treatments. This is a measure of clustering of malaria infections	$\uptau \ge 1$	Estimated from cross-sectional survey data

The equation for the number of infectious individuals, $I_{t}$, for the simple SIS model [10] in the absence of intervention is

1 $$\begin{aligned} I_{t + 1} =\, & \frac{\beta }{N}I_{t} \left( {N - I_{t} } \right) + \gamma I_{t} \\ =\, & \left( {\beta + \gamma } \right) I_{t} - \frac{\beta }{N}I_{t}^{2} . \\ \end{aligned}$$

The fixed points (equilibria) of this model are the solutions of

$$I = \left( {\beta + \gamma } \right) I - \frac{\beta }{N}I^{2} ,$$

which is equivalent to:

$$I\left( {\frac{\beta }{N}I + 1 - \left( {\beta + \gamma } \right)} \right) = 0 .$$

This quadratic equation has two solutions, the disease-free (trivial) equilibrium, $I_{dfe} = 0$, and the endemic (positive) equilibrium point,

$$\begin{aligned} I^{ *} =\, & \frac{\beta + \gamma - 1}{\beta /N} \\ =\, & \frac{{N\left( {\beta + \gamma - 1} \right)}}{\beta } . \\ \end{aligned}$$

The stability of $I_{dfe}$ is determined by differentiating the right-hand side of Eq. (1) with respect to $I$ at the fixed point,

$$\frac{\partial }{\partial I}\left. {\left( {\left( {\beta + \gamma } \right) I - \frac{\beta }{N}I^{2} } \right)} \right|_{I = 0} = \beta + \gamma ,$$

implying that $I_{dfe} = 0$ is locally asymptotically stable if $\beta + \gamma < 1$, and unstable if $\beta + \gamma > 1$. Furthermore, $I^{*}$ exists ($I^{*} > 0$) if and only if $\beta + \gamma > 1$. Therefore, $\beta + \gamma > 1$ is the condition for transmission to be sustained. By definition, $\gamma < 1$ so $\frac{\beta + \gamma - 1}{\beta } < 1$ and $I^{*} < N$. This, however, does not guarantee the stability of $I^{*}$, which could be unstable with the existence of other attractors for certain regions of the parameter space.

The value of the basic reproduction number is

2 $$R_{0} = \frac{\beta }{1 - \gamma }.$$

### Models of reactive case detection

The RCD programme is assumed to be added to a routine case management system, which serves as a passive surveillance component of a surveillance-response system. The RCD functions by investigating a constant number of passively detected index cases per unit time ($\upiota$), and testing and treating a constant number ($\upnu$) of neighbours of these index cases. The effect of the system on the controlled reproduction number, *R*_*c*_, is then determined by $\upiota$, $\upnu$ and the targeting ratio ($\uptau$), which is equivalent to the ratio of the prevalence of infection in the neighbourhood of an index case to the prevalence in the general population. The coverage of RCD, defined as the proportion of all infections detected, in unit time, is then: $\varPhi = \frac{{\upiota \upnu \uptau }}{\text{N}};$ the proportion of individuals tested that is positive (assuming a perfect diagnostic) is $\tau p$ (where $p$ is the point prevalence of the general population), and the number of infections detected (and treated) per unit time is $\varphi = Np\varPhi =\upiota \upnu \uptau p$, and the proportion that escape detection (the escape probability) $\varepsilon = 1 - \varPhi$.

RCD is included in the general SIS model by assuming both the standing crop of infections (or equivalently the infectious reservoir contributing new infections), is reduced by $\varphi_{t}$, the number of infections detected (and treated) per unit time. Thus:

3 $$I_{t + 1} = \frac{\beta }{N}\left( {I_{t} - { \hbox{min} }\left( {\varphi_{t} ,I_{t} } \right)} \right)\left( {N - I_{t} } \right) + \gamma \left( {I_{t} - { \hbox{min} }\left( {\varphi_{t} ,I_{t} } \right)} \right),$$

where the minimum function is required to ensure that the number of infections, $I_{t} \ge 0$ (necessary for model (b) below). Three different models (a–c below) for reactive case detection and the particular form for $\varphi_{t}$ were implemented.

#### SIS model with constant targeting ratio ($\tau$)

The model for RCD with the simplest dynamics assumes a constant value for $\uptau$ that is independent of prevalence. This is a strong simplifying assumption which results in a model that is equivalent to repeated regular deployments of mass test and treat at coverage levels amplified by $\uptau$, so that

$$\varphi_{t} \left( {I_{t} } \right) =\uptau \upiota \upnu p =\uptau \upiota \upnu I_{t} /N.$$

Equation 3 then becomes

$$I_{t + 1} = \frac{\beta }{N}\left( {I_{t} - { \hbox{min} }\left( {\uptau \upiota \upnu I_{t} /N,I_{t} } \right)} \right)\left( {N - I_{t} } \right) + \gamma \left( {I_{t} - { \hbox{min} }\left( {\uptau \upiota \upnu I_{t} /N,I_{t} } \right)} \right).$$

If $\uptau \upiota \upnu \ge N$, then $I_{t + 1} = 0$, irrespective of $I_{t}$ (corresponding to the reactive component immediately finding all the infections because the amplified coverage is greater than the population size) and the disease-free (trivial) equilibrium, $I_{dfe} = 0$ is achieved within one time-step. If $\uptau \upiota \upnu < N$ then the effect of the RCD programme is to multiply both $\upbeta$ and $\gamma$ by the constant $\varepsilon = 1 -\uptau \upiota \upnu /N$ (the proportion of infections that escape treatment in any one time step) so that

$$I_{t + 1} = \varepsilon \left( {\frac{{\beta I_{t} }}{N}\left( {N - I_{t} } \right) + \gamma I_{t} } \right),$$

leading to the endemic equilibrium,

$$I^{ *} = \frac{{N\left( {\beta + \gamma - 1/\varepsilon } \right)}}{\beta } ,$$

and controlled reproduction number,

$$R_{c} = \frac{\beta }{1/\varepsilon - \gamma },$$

implying that $I_{dfe} = 0$ is locally asymptotically stable if $\beta + \gamma < 1/\varepsilon$, and unstable if $\beta + \gamma > 1/\varepsilon$, so $\beta + \gamma > 1/\varepsilon$ is the condition for transmission to be sustained. Correspondingly, the condition for the RCD programme to achieve elimination can be expressed as a function of $R_{0}$ by substituting from Eq. 2. This gives a critical value of $\varepsilon$ of

$$\varepsilon^{ *} = \frac{1}{{R_{0} - \gamma R_{0} + \gamma }} .$$

#### SIS model with varying targeting ratio ($\tau$) but constant treatment rate ($\varphi$)

An alternative to assuming $\uptau$ to be constant is to assume a constant value of $\varphi_{t}$ that does not depend on prevalence. This corresponds to the situation where the performance of the RCD programme is limited because of fixed capacity to diagnose and treat infections. This may approximate reality if staffing levels or the unit cost of treatments determine throughput. Therefore Eq. (3) remains as is, and $\varphi_{t}$ becomes a model parameter, $\varphi$.

$$I_{t + 1} = \frac{\beta }{N}\left( {I_{t} - { \hbox{min} }\left( {\varphi ,I_{t} } \right)} \right)\left( {N - I_{t} } \right) + \gamma \left( {I_{t} - { \hbox{min} }\left( {\varphi ,I_{t} } \right)} \right).$$

It follows that, if $I_{t} \le \varphi$ (when the follow up capacity of the programme is greater than the number of infected individuals in the population), then $I_{t + 1} = 0$, hence $I_{dfe} = 0$,If $I_{t} > \varphi$,

4 $$I_{t + 1} = \frac{\beta }{N}\left( {I_{t} - \varphi } \right)\left( {N - I_{t} } \right) + \gamma \left( {I_{t} - \varphi } \right).$$

In contrast to the model with constant $\uptau$, the escape probability varies in the course of the RCD programme, since

$$\upvarepsilon_{t} = 1 - \frac{{\upiota \upnu \uptau _{t} }}{N} = 1 - \frac{\varphi }{{I_{t} }}.$$

It follows that the escape probability increases with $I_{t}$, and in particular, that it is very low if $I_{t}$ is small, so the endpoint achieved may depend on the initial prevalence.

The fixed points of this system are the solutions of

$$I = - \frac{\beta }{N}I^{2} + \beta I + \frac{\beta \varphi }{N}I + \gamma I - \left( {\beta + \gamma } \right)\varphi ,$$

$$0 = \frac{\beta }{N}I^{2} + \left( {1 - \left( {\beta + \gamma } \right) - \frac{\beta \varphi }{N}} \right)I + \left( {\beta + \gamma } \right)\varphi .$$

These are determined by the ranges of $I_{t}$ within $\varphi \le I_{t} \le N$ where the right-hand side of Eq. 4 is constant or decreasing, conditional on $R_{0} > 1$, (and hence $\beta + \gamma > 1$ from Eq. 2). There are three equilibria satisfying these conditions, namely the disease-free equilibrium point, $I_{dfe} = 0$, and two endemic equilibria:

$$I^{ *} = \frac{{N\left( {\beta + \gamma - 1} \right) + \beta \varphi \pm \sqrt {\left( {N\left( {\beta + \gamma - 1} \right) + \beta \varphi } \right)^{2} - 4N\left( {\beta + \gamma } \right)\beta \varphi } }}{2\beta },$$

which exist if and only if $\left( {N\left( {\beta + \gamma - 1} \right) + \beta \varphi } \right)^{2} > 4N\left( {\beta + \gamma } \right)\beta \varphi$.

The stability of the fixed points is not analytically derived here, but explored through numerical simulations and bifurcation analysis.

#### SIS model with varying targeting ratio ($\tau$) and treatment rate $\left( \varphi \right)$

More generally, both $\tau$ and $\varphi_{t}$ might vary over time because infections become harder to find as prevalence decreases (leading to a decrease in $\varphi_{t}$) while targeting is a priori likely to improve as transmission becomes more localized. $\varphi_{t}$ should, therefore, vary dynamically with $p = \frac{{I_{t} }}{N}$, so that:

$$\varphi_{t} = \iota \nu \frac{{I_{t} }}{N}\tau \left( {\nu ,p} \right),$$

where $\tau \left( {\nu ,p} \right)$ is a smooth function of *p* and of ν satisfying the constraints $\tau \left( {\nu ,1} \right) = 1$ (since targeting achieves nothing if everyone is infected) and $\tau \left( {N,p} \right) = 1$ (since there can be no targeting if the whole population is tested). Various functional relationships satisfying these constraints, were explored and the one giving the best fit to the observed relationships between the numbers of infections found in a field study conducted in Zambia, as described below, and the numbers of individuals tested is described in Additional file 1.

The escape probability again varies over time and is

$$\upvarepsilon_{t} = 1 - \frac{{\upiota \upnu \uptau _{t} }}{N} = 1 - \frac{{\varphi_{t} }}{{I_{t} }}.$$

Analytical determination of the fixed points of this model is not feasible, so the fixed points and their stability was explored numerically.

### Estimating the targeting ratio from the field data

To parameterize model (c), the function $\tau \left( {\nu ,p} \right)$ was estimated from the Zambian field data. Data from the censuses carried out for a cluster-randomized trial of mass drug administration (MDA) and focal MDA (fMDA) in 60 health facility catchment areas in Southern Province, Zambia [11] were used to estimate this function.

At the start of the trial, a total population of 212,049 individuals were censused and their households geolocated. The MDA involved two rounds of treatment with dihydroartemisinin–piperaquine (DHAP) with 88.1% and 72.0% coverage, respectively. The fMDA arms entailed testing (with a histidine-rich protein II (HRP-2) based rapid diagnostic test (RDT)) for *Plasmodium falciparum* and treating all members of households in which any individuals had positive tests, and achieved 62.5% and 54.0% coverage in the two distinct rounds [11]. These interventions were in addition to the activities of community health workers and high levels of access to anti-malarial treatment at health facilities [11].

Two cross sectional parasitological surveys were carried out, one before the intervention in May 2014 (941 of 3036 individuals tested positive by RDT), and one afterwards in May 2015 (176 of 2107 individuals tested positive by RDT). Estimates of how $\uptau$ should depend on $\upnu$ and $p$ were based on analyses of these surveys (Additional file 1), assuming that the clustering of positivity is independent of the diagnostic sensitivity. More general results of these surveys and of the intervention impacts in the trial are presented elsewhere [11].

## Results

### SIS model

**Fig. 1:**
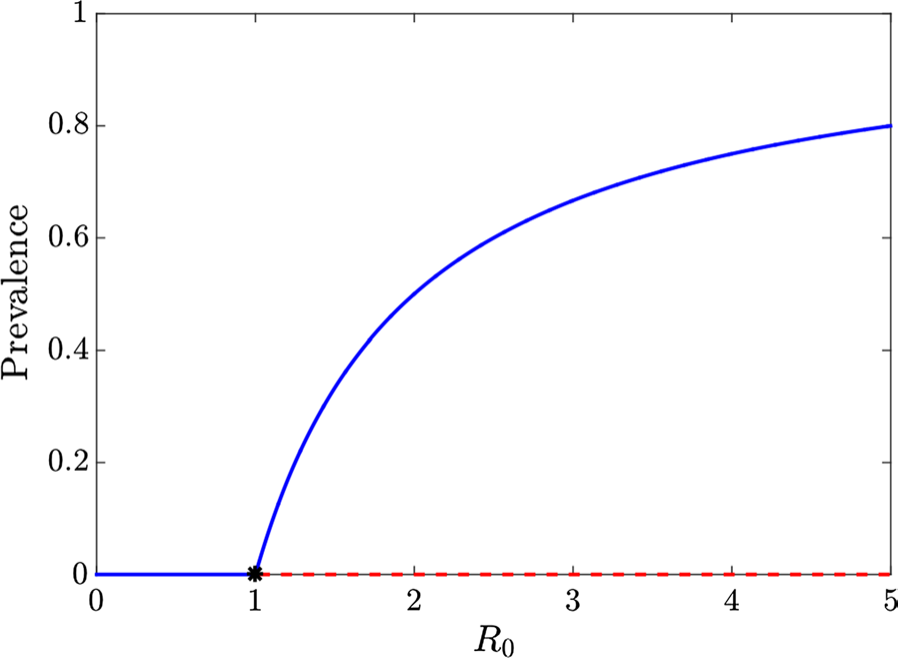
Bifurcation diagram showing fixed points of prevalence of the simple SIS model. The solid blue lines correspond to locally asymptotically stable equilibria. The dashed red lines correspond to unstable equilibria. The disease-free equilibrium point is locally asymptotically stable for $R_{0} \le 1$ and unstable for $R_{0} > 1$ (where a transcritical bifurcation occurs at $R_{0} = 1$). The endemic equilibrium point is locally asymptotically stable for $R_{0} > 1$. Here the infectious period is assumed to be 200 days and the transmission parameter, $\beta$, is varied to provide the appropriate value of $R_{0}$

The SIS transmission model (1) captures the most immediately relevant characteristics of malaria transmission in humans in settings where elimination might be considered. In this model there is a stable endemic level of prevalence, which is a monotonic function of $R_{0} ,$ for all but very high values of $R_{0}$ (Fig. 1) (For values of approximately $R_{0} > 57$, the endemic equilibrium goes through a period doubling bifurcation that eventually leads to chaotic dynamics as has been shown for the logistic difference equation model [17]).

Since high values of $R_{0}$ are not relevant for this analysis, $R_{0}$ is assumed to remain below this threshold so the endemic equilibrium point is globally asymptotically stable for $R_{0} > 1$ and any positive initial prevalence will approach the endemic steady state prevalence—the solid blue line in Fig. 1. (Prevalence increases over time if the initial prevalence is lower than the endemic prevalence and decreases if the initial prevalence is higher than the endemic prevalence.) In transmission settings with $R_{0} \le 1$, any positive initial prevalence will monotonically decrease towards zero over time (implying also protection against sporadic importation).

### Models of RCD

The reduction in the steady state prevalence that is achieved by the RCD, the fixed points and their stability (hence the circumstances determining whether transmission can be eliminated with RCD), and the time that the programme takes to reach the steady state, depend on the formulation of the effect of RCD.

**Fig. 2:**
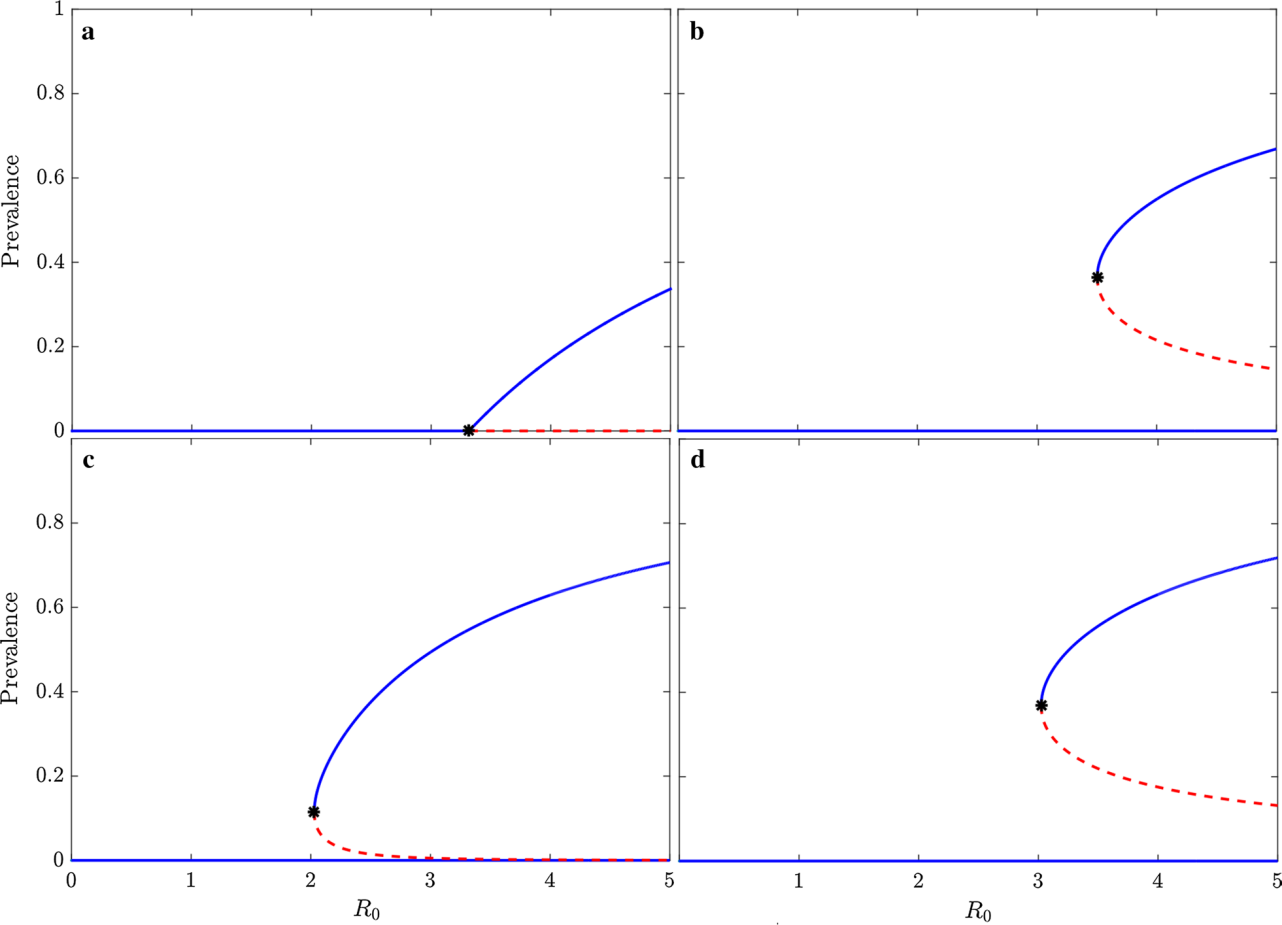
Bifurcation diagrams showing fixed points of prevalence for RCD models. The solid blue lines correspond to locally asymptotically stable equilibria. The dashed red lines to unstable equilibria. The infectious period for all models is set to 200 days and the transmission parameter, $\beta$, is varied to provide the appropriate value of $R_{0}$. **a** Model (a): $\uptau = 5$, weekly total number of neighbours tested (product of $\upiota$ and $\upnu$) is 150. The disease-free equilibrium point is locally asymptotically stable for $R_{0}$ less than a critical value and unstable above this value (where a transcritical bifurcation occurs). The endemic equilibrium point is locally asymptotically stable for $R_{0}$ greater than the critical value. **b** Model (b): $\varphi = 150$. The disease-free equilibrium point is locally asymptotically stable for any value of $R_{0}$. There are two endemic equilibria for values of $R_{0}$ greater than a certain threshold (where a saddle node bifurcation occurs). The larger endemic equilibrium point is locally asymptotically stable and the smaller endemic equilibrium point is unstable. **c** Model (c): $\upiota = 3$, $\upnu = 50$; the targeting ratio,$\uptau$, is calculated for the endemic prevalence in the absence of RCD. The disease-free equilibrium point is locally asymptotically stable for any value of $R_{0}$. There are two endemic equilibria for values of $R_{0}$ greater than a certain threshold (where a saddle node bifurcation occurs). The larger endemic equilibrium point is locally asymptotically stable and the smaller endemic equilibrium point is unstable. **d** Model (c): $\upiota = 50$, $\upnu = 3$; $\uptau$ is calculated for the endemic prevalence in the absence of RCD. the disease-free equilibrium point is locally asymptotically stable for any value of $R_{0}$. There are two endemic equilibria for values of $R_{0}$ greater than a certain threshold (where a saddle node bifurcation occurs). The larger endemic equilibrium point is locally asymptotically stable and the smaller endemic equilibrium point is unstable

For model (a) of RCD with a constant targeting ratio, $\tau$, RCD is able to eliminate transmission in settings where $R_{0} > 1$ but does not qualitatively change the asymptotic dynamics of the system, as seen in the bifurcation diagram in Fig. 2a. The exact threshold value of $R_{0}$ where the transcritical bifurcation occurs (stable endemic transmission is possible) depends on the assumed value of the targeting ratio and the number of people tested weekly by the RCD programme (the product of the number of index cases and the neighbours followed per index case).

For the models of RCD with varying τ, but constant $\varphi$ (model b), there is a qualitative change in the asymptotic dynamics of the system, as shown in the bifurcation diagram in Fig. 2b. The lower branch of unstable endemic equilibrium points divides the basins of attraction of the disease-free equilibrium point and the larger endemic equilibrium points. If the prevalence of infection is less than this unstable equilibrium point, then the disease will die out and RCD will lead to elimination. If the prevalence is greater than the unstable endemic equilibrium point, then the system will asymptotically approach the larger endemic equilibrium point and reactive case detection will be insufficient to eliminate transmission on its own. As the coverage of RCD increases, the two branches of endemic equilibrium points move to the right and compress towards the centre (the branch of larger endemic equilibrium points moves down and the branch of smaller endemic equilibrium points moves up). Therefore, the region where reactive case detection is sufficient to not allow any transmission to occur is greater. For values of $R_0$ where transmission can occur, the stable endemic prevalence in the presence of reactive case detection is lower, and the threshold prevalence below which RCD can eliminate transmission is higher.

For the models with varying τ and varying $\varphi$ the bifurcation diagrams are shown in Fig. 2c and d. Similar to Fig. 2b, the lower branch of endemic equilibrium points divides the basins of attraction of the disease-free equilibrium point and the larger endemic equilibrium points. If the prevalence of infection is less than this unstable equilibrium point, then the disease will die out and RCD will lead to elimination. If the prevalence is greater than the unstable endemic equilibrium point, then the system will asymptotically approach the larger endemic equilibrium point and reactive case detection will be insufficient to eliminate transmission on its own.

Figure 2c shows the bifurcation diagram for following 3 index cases and investigating 50 neighbours, while Fig. 2d the diagram for following 50 index cases and investigating 3 neighbours each. These extreme cases shows that the values of $\upiota$ and $\upnu$ affect the equilibrium points, even if the total neighbours investigated are the same. In both cases, the branches corresponding to endemic equilibria have moved to the left when compared to Fig. 2b. This implies that in the (more plausible) model where number of cases detected by RCD depends on prevalence, RCD can only lead to elimination in a lower range of transmission settings. Furthermore, the branches of unstable equilibria have also shifted down implying there is also a lower threshold of prevalence where RCD can lead to elimination.

Since the branch of equilibrium points in Fig. 2d is further to the right of that in Fig. 2c, RCD with these characteristics is able to eliminate and maintain elimination for a much larger range of $R_{0}$. For high values of $R_{0}$ the dashed red line in Fig. 2d is also higher than that in Fig. 2c implying that RCD can maintain elimination for a higher importation rate (or at a higher starting prevalence) when there are many index cases and testing fewer people per index case than vice versa. One version of this strategy would be to follow as many people as possible and only test household members.

### Transient dynamics

**Fig. 3:**
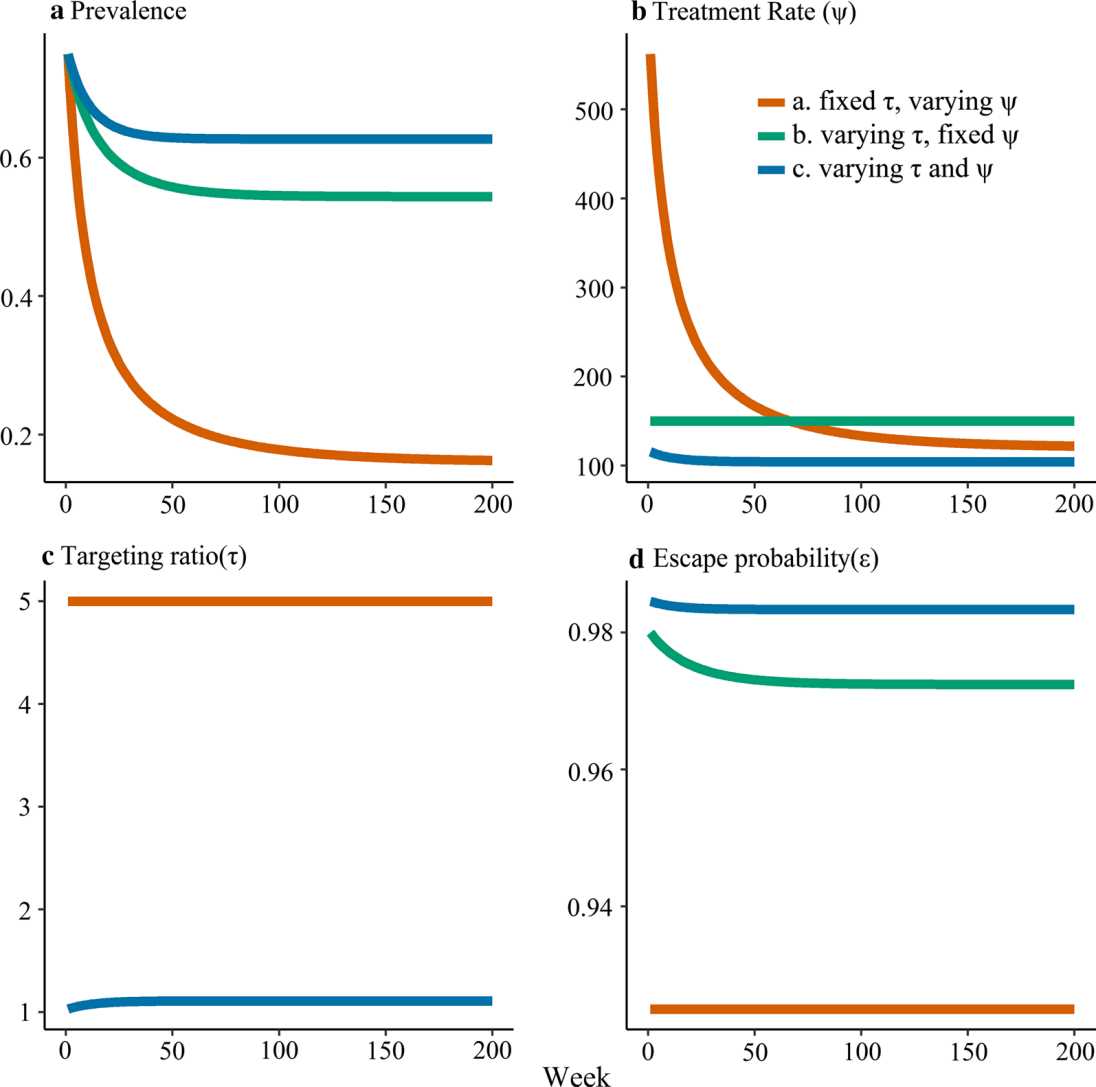
Time course for SIS models of RCD with $\varvec{R}_{0} = 4$ showing **a** Prevalence; **b** Treatent rate; **c** Targeting ratio; and **d** Escape probability. The models were initialized at the non-intervention endemic steady state for parameter values corresponding to $R_{0} = 4$, the infectious period is assumed to be 200 days and the transmission parameter, $\beta$, corresponds to $R_{0} = 4$. The RCD parameter values are for model (a): $\uptau = 5$ and $\upiota \upnu = 150$ (corresponding to parameter values in Fig. 2a); for model (b): $\varphi = 150$ (corresponding to parameter values in Fig. 2b); for model (c): $\upiota = 10$ and $\upnu = 15$ (intermediate between the parameter values in Fig. 2c and d). The targeting ratio is not defined for model (b) so is not presented in **c**. The simulation results show that in high transmission settings, the more realistic model for RCD (c) only leads to a small reduction in prevalence since the targeting ratio remains close to 1

**Fig. 4:**
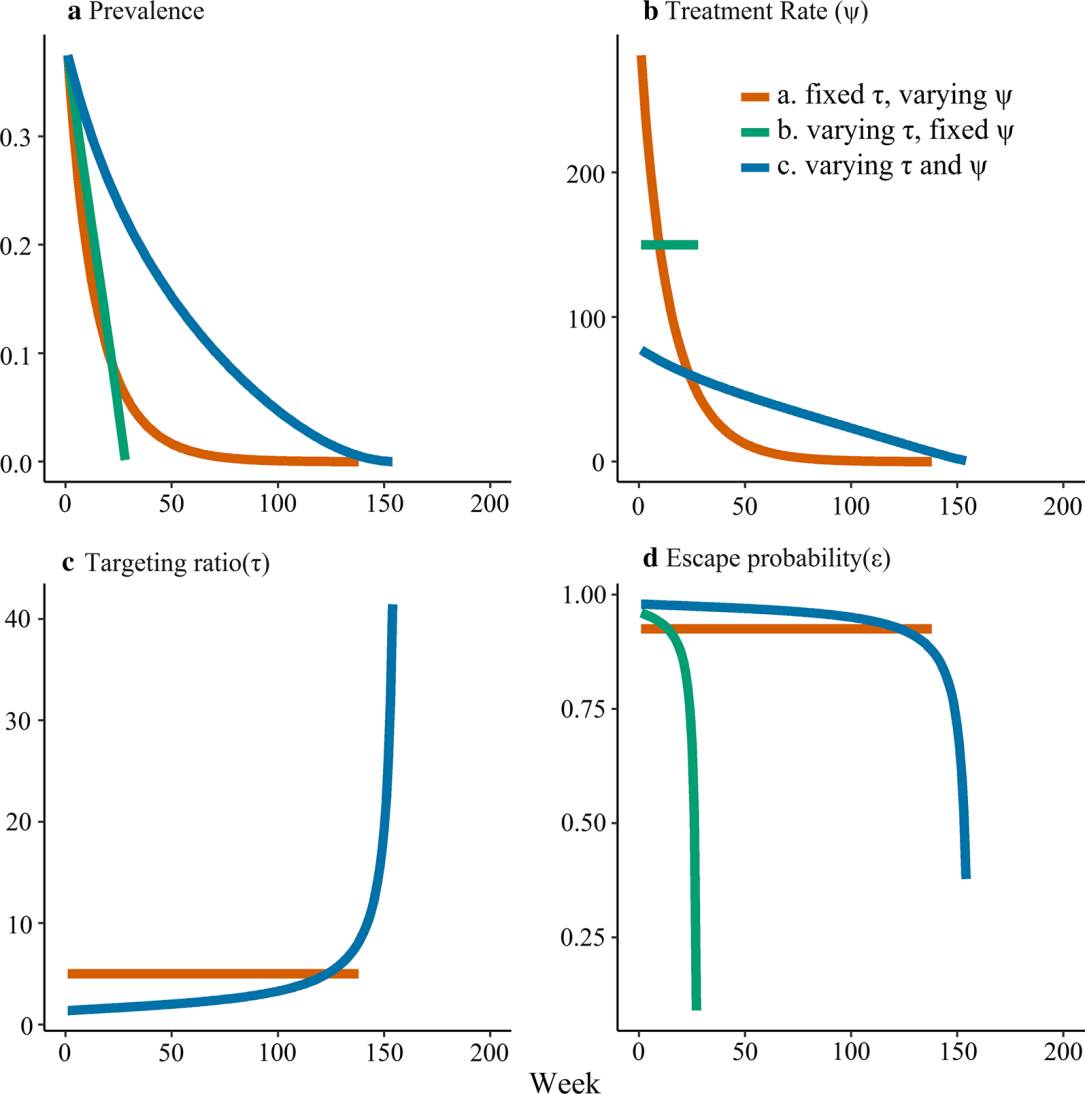
Time course for SIS models of RCD with low ${R}_{0}$ showing **a** Prevalence; **b** Treatent rate; **c** Targeting ratio; and **d** Escape probability. The models were initialized at the non-intervention endemic steady state for parameter values corresponding to ${R}_{0} = 1.6$. All parameters other than $\upbeta$ are as in Fig. 3

Figures 3 and 4 show the transient dynamics of RCD for all three models for two transmission settings assuming that initial prevalence is at the (pre-RCD) endemic equilibrium. If the transmission potential is high, where none of the three models predict extinction, the prevalence adjusts rapidly to a new endemic equilibrium (Fig. 3a). Simultaneously, the treatment rate, $\varphi$ (Fig. 3b), and the targeting ratio, τ (Fig. 3c), also rapidly adjust. The escape probability, $\epsilon$ (Fig. 3d) remains close to 1 in these scenarios since most infections are not treated. When the transmission potential is lower, so that extinction occurs in all three models, this happens quickly for models (a) and (b) but more slowly for model (c) (Fig. 4a). Similarly, the treatment rate adjusts rapidly for model (a) (Fig. 4b) and the escape probability adjusts rapidly for model (b) (Fig. 4d), while the treatment rate, targeting ratio and escape probability adjust slowly for model (c) (Fig. 4b–d).

For model (a) with constant τ, as prevalence quickly declines to the new steady state (Figs. 3a and 4a), $\varphi$ adjusts downwards to a new value (Figs. 3b and 4b), but the escape probability remains constant (Figs. 3d and 4d), since the asymptotic dynamics of this system are similar to those of the simple SIS model in Fig. 1.

Since model (b) removes a constant number of infected individuals regardless of the prevalence level, the escape probability decreases as prevalence decreases leading to positive feedback, so RCD either causes the system to reach a new endemic prevalence when the transmission setting is high (Fig. 3) or elimination when the transmission setting is low and prevalence and the escape probability drop rapidly (Fig. 4).

In the high transmission setting where model (c) does not predict elimination, prevalence quickly approaches the new endemic equilibrium (which is only slightly lower) and there is little change in the number of people tested, the targeting ratio or the escape probability (Fig. 3). In the low transmission scenario where model (c) predicts extinction, prevalence first declines gradually, with a concomitant increase in targeting ratio. The positive feedback loop in this model not only leads to an i ncrease in the targeting ratio, but also in the number of people treated leading to a sharp decrease in the escape probability and consequently with a faster than exponential decrease in the prevalence (Fig. 4). This is particularly clear in Fig. 4a, where the curve for the prevalence has an inflection point, where it is initially convex at higher prevalence, but becomes concave at lower prevalence before quickly approaching zero.

**Fig. 5:**
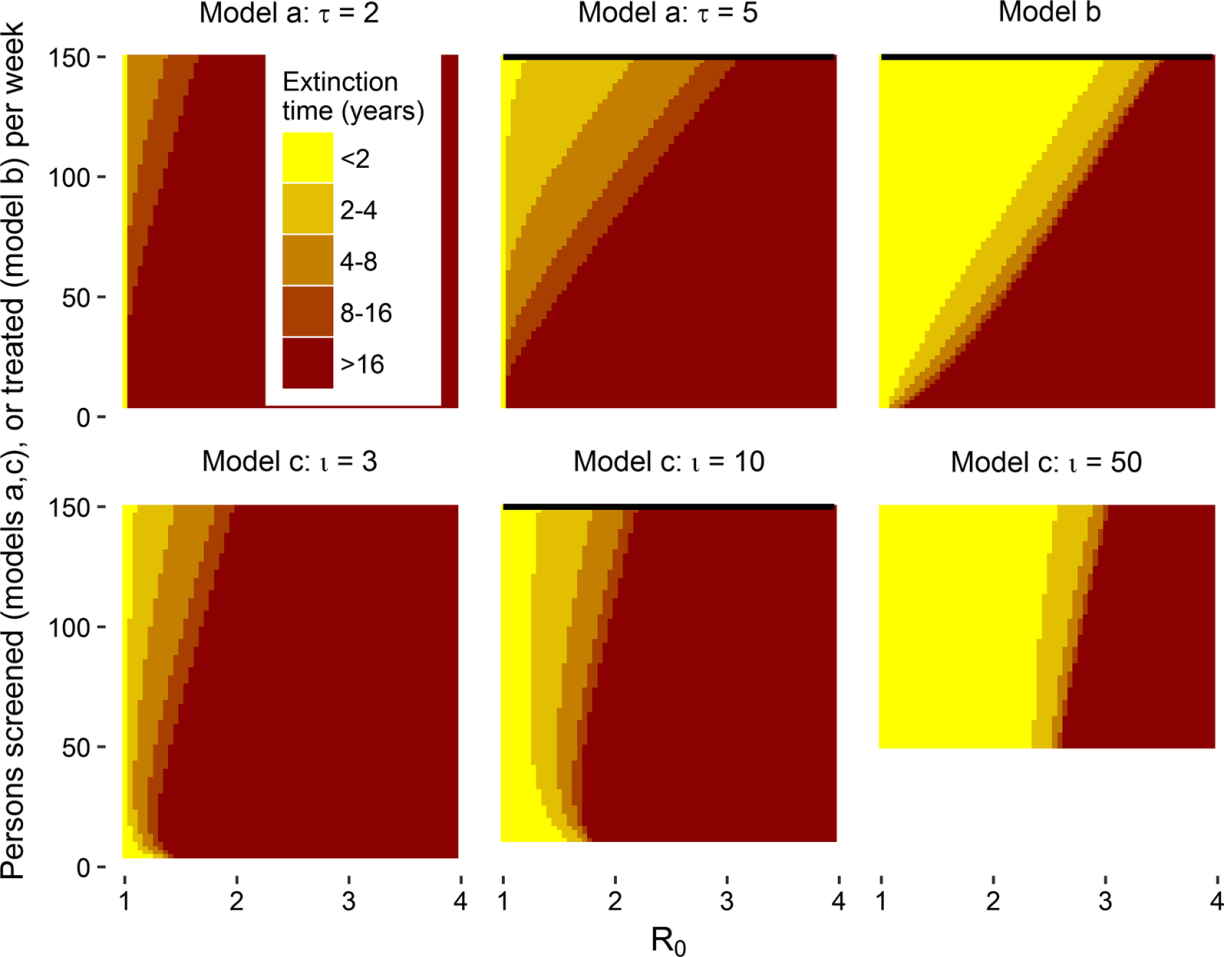
Time to extinction in SIS models of RCD. Since the programme must screen at least one person for each index case, the function is shown only for $\upnu > \iota$. The infectious period is assumed to be 200 days; transmission parameter is chosen to corresponding to the value of $R_{0}$ shown on the x-axis; and the RCD parameters are described in the sub-figure titles and y-axis. The black lines indicate the parameter sets illustrated in Figs. 3 and 4. In model (c) the lack of monotonicity in time to extinction as a function of $R_{0}$ arises because the function used for $\tau \left( {\upnu,{\text{p}}} \right)$ does not strictly enforce a requirement that $\psi$ increases with $\upnu$

Figure 5 shows the time to elimination, [where elimination is defined as zero prevalence for models (b) and (c) and prevalence < 0.01% for model (a)], for the three models for different parameter settings. Figure 5 suggests that for models (a) and (b), the time to elimination and the likelihood of elimination depends on both, the transmission setting and the operational characteristics of the RCD programme. Furthermore, while elimination may be somewhat gradual for model (a), it either occurs rapidly or not at all for model (b). However, for the more realistic model of RCD [model (c)], the likelihood of elimination depends strongly on the transmission setting but not on the operational parameters of RCD, suggesting, that in the settings where RCD would be sufficient to eliminate transmission, it would work even if coverage is low. For most settings where RCD is insufficient to eliminate transmission, improving the coverage of RCD would not necessarily lead to better outcomes.

## Discussion

SIS models can capture the most relevant characteristics of malaria transmission in humans in settings where elimination might be considered; in particular a stable endemic level of prevalence is achieved which is a monotonic function of $R_{0}$ (over the relatively low values of $R_{0}$ where elimination is feasible).

It makes the main conclusions clear by using a simple approximation for malaria transmission, which ignores factors such as super-infection and acquired immunity that are mostly relevant to high transmission settings where RCD would not be considered. Other simplifications are the lack of seasonality and heterogeneity, which would be important for quantitative predictions about any specific setting, but which should not affect the general principles and qualitative understanding of RCD derived here. In settings where transmission alternates between low and very low, seasonality may reduce the effectiveness of RCD during the very low transmission season towards that of the low transmission season (since there is less seasonal variation in prevalence). Programmes in higher transmission settings are unlikely to consider RCD and seasonal malaria chemoprevention may be a more appropriate intervention when relatively high transmission is concentrated in one season.

The simple models also did not include the sensitivity of diagnostic tests so could not distinguish between RCD and other reactive interventions as fMDA. The models here are more representative of fMDA, which was indeed found to be more effective than RCD from a study in Zambia [18].

Although the models did not explicitly consider continuous importation (which would be included by a constant positive term, independent of $I_{t}$, in the right hand side of Eq. (1), the bifurcation diagrams provide insight on the impact of irregular importation (as is likely to happen in reality).

The three formulations (a–c) differ substantially in the results of the stability analysis. Model (a) has qualitatively similar asymptotic dynamics to the baseline SIS model although RCD is able to achieve and maintain the disease free status for some values of $R_{0}$ greater than 1, and reduces the endemic prevalence for values of $R_{0}$ above this threshold—although the quantitative impact depends on the parameter values chosen. Models (b) and (c) show qualitatively different dynamics to the SIS model and model (a), usually not seen in models of infectious diseases, where RCD is able to maintain a locally asymptotically stable disease-free state for any value of $R_{0}$. However, the basin of attraction (the prevalence level where infection would die out and the system would return to the disease-free state) decreases as $R_{0}$ increases—so at high transmission levels, RCD can only maintain elimination if the importation pressure is very low. There is also a threshold value of $R_{0}$ greater than 1 (where a saddle node bifurcation occurs), below which transmission cannot be maintained, and above which two endemic equilibria co-exist, with the unstable lower equilibrium point separating the basins of attraction of the stable endemic equilibrium point and the stable disease-free equilibrium point. For any value of $R_{0}$ over this threshold, if a transient strategy such as mass drug administration or intensive vector control can reduce prevalence below the unstable lower equilibrium point (below the dashed red line in Fig. 2b–d), RCD would then be able to push the system to elimination. Therefore, the higher this unstable equilibrium is (and closer to the stable equilibrium—the solid blue line in Fig. 2b–d), the more likely that supplementing RCD with an additional potentially transient prevalence reducing intervention would lead to elimination. Furthermore, the stable endemic equilibrium prevalence for a particular value of $R_{0}$ is also lower than the corresponding prevalence for the SIS model, so where RCD does not lead to elimination, it reduces the endemic prevalence.

This behaviour is markedly different from the backward bifurcation often seen in many infectious disease (and in particular malaria) models, although both exhibit saddle node bifurcations and the bifurcation diagrams may look somewhat similar. In these models, a transcritical bifurcation occurs at $R_{0} = 1$ so the disease-free endemic equilibrium loses stability at $R_{0} = 1$ and transmission is endemic above this value. Furthermore, the saddle node bifurcation occurs at a value of $R_{0}$ less than 1, so transmission is always possible for $R_{0} > 1$ and even possible for some values of $R_{0} < 1$.

Model (c), where the targeting ratio, $\tau$, is allowed to vary with prevalence and the search radius, does not reduce the stable endemic equilibrium prevalence as much as model (b) so in this more realistic model, RCD is not as effective as the simplified model of RCD.

Furthermore, the unstable equilibrium point for model (c) is also lower than that for model (b) so the range of prevalence levels where RCD is sufficient to eliminate transmission or to prevent reintroduction is also not as great as for the simplified model. Further analysis of model (c) suggests that following more index cases and testing fewer neighbours is more effective than following fewer index cases and testing many neighbours because the targeting ratio is much higher over short distances (at least in the Zambian dataset analyzed here [12]). Even though malaria transmission is mediated by mosquitoes that frequently travel several hundred metres, people in the immediate vicinity of an infected person are much more likely to be infected than the average in the population, presumably because of shared risk-factors. Over distances of tens or hundreds of metres there is rather little to be gained by targeting. This is coherent with analyses of the same data from Zambia that suggest that the impact of RCD with larger search ratios is limited except when prevalence is very low [12], and with a recent analysis of the RCD programme in eSwatini in which screening was carried out over radii of 500 m of index cases, 26.7% of infections were found in the same household as the index case and a further 41% within 100 m [19].

Analysis of transient behaviour of model (c) suggests that the likelihood of elimination and time to elimination depends mostly on the transmission setting and the operational characteristics of the RCD programme (number of index cases followed and neighbours tested) do have a large impact. Therefore, settings where RCD is implemented for elimination should be identified carefully.

In general, the simplest reactive strategy is treatment of passively detected cases, and previous simulations of a stochastic individual based simulation model, *OpenMalaria*, indicate that if importation rates are low and transmission potential is reduced (for instance by vector control) [20], high coverage of case management can reliably prevent re-establishment of *P. falciparum* transmission if it is interrupted. This can be considered as a limiting case in which resources allocated to testing or treating uninfected people are minimized. RCD goes beyond any possible improvement in passive case management coverage by potentially addressing asymptomatic infections, or catching new infections just as they start to become symptomatic. For a fixed testing capacity, RCD should aim to achieve a high specificity since each encounter with an uninfected person reduces programme efficiency. Supplementing passive case detection with treatment of other household members (and perhaps their immediate neighbours) is one operationally attractive way of doing this because the other household members can more easily be located, and even simple strategies like dispensing a single dose of drugs, such as sulfadoxine-pyrimethamine for each family member, might be feasible.

The analysis of the three model formulations (a–c) helps to clarify what might be the most efficient way to optimize RCD. The limited range of settings where model (a) interrupts transmission, suggests that improved technologies for targeting around index cases (e.g. by profiling to identify high-risk individuals) would not, on their own, have a big impact on the range of settings where transmission can be interrupted. Front-loading an RCD programme by searching a very wide radius until a target number of infections are found each time period [the very resource-demanding process simulated by model (b)] may speed up interruption of transmission but will be successful in fewer settings than the strategy of model (c) with constant search intensity, corresponding most closely to existing practice. Front-loading will make little difference to the total number of treatments required where transmission is interrupted, which is mainly a function of $R_{0}$ and does not depend very much on the screening intensity or strategy also suggesting that as prevalence decreases, a feedback loop would occur so that after a “tipping point” could quickly lead to elimination, serving as a possible example of “accelerating to zero”.

Where RCD is effective, its effects depend largely on the targeting ratio. This is a measurable quantity, and can be determined from basic parasitological survey data even in the absence of a programme, so this could be part of a feasibility study before any commitment is made to implementing RCD. Alternatively (and better) data to parameterize the model could be obtained directly from programmes already implementing targeted case searches, providing there is some way of estimating the overall prevalence in the community. A meta-analysis four studies of RCD gave an average estimate of $\tau$ of 5.3 (95% CI 3.3, 8.5) for searches in the immediate vicinity of the case [21]. This is rather close to the value we would expect for a search of only immediate household members in the Zambian site. It is possible that the values of this quantity do not vary very much across sites, and it is reasonable to treat this as a best-case estimate for the model with constant $\tau$. The targeting ratio measured in the same way, can also be used to design reactive vector control strategies, which may have greater impact on $R_{c}$ and hence on the chances of eliminating the parasite.

## Conclusion

The analyses here suggest that elimination of malaria can be achieved via RCD alone in only a very limited range of settings where transmission potential is very low, and improving the coverage of RCD has little effect on this range. RCD may also help maintain the disease-free state in the face of imported infections. In a rather wider range of settings it is likely to reduce disease burden and achieve new steady state endemicities. The reduction depends strongly on the targeting ratio, which can be estimated from prevalence survey data. Where the endemicity is reduced this may make it more feasible to achieve elimination by adding in further interventions. Conversely scaling up of other interventions such as LLINs might reduce transmission to levels where RCD could achieve elimination. Further analyses of these models can be used to examine the synergies of non-targeted interventions with RCD, indicating the most efficient ways to complement it.

## Additional file

**Additional file 1. ** Parameterisation of the models using Zambian field data.

## Data Availability

The data and underlying code are available on request from the authors.

## Abbreviations

DHAP: dihydroartemisinin–piperaquine
fMDA: focal mass drug administration
HRP-2: histidine-rich protein II
LLINs: long-lasting insecticidal nets
MDA: mass drug administration
RCD: reactive case detection
RDT: rapid diagnostic test
SIS: susceptible-infectious-susceptible
