# Supplementary material for: Theory of reactive interventions in the elimination and control of malaria
Source: Malar J. 2019 Aug 2;18:266. doi: 10.1186/s12936-019-2882-z (PMC6679501; doi:10.1186/s12936-019-2882-z)
Supplement: Supplementary file 1 — Additional file 1. Parameterisation of the models using Zambian field data. [file 12936_2019_2882_MOESM1_ESM.docx]

# Supplementary Information to:

# Theory of reactive interventions in the elimination and control of malaria

Nakul Chitnis, Peter Pemberton-Ross, Josh Yukich, Busiku Hamainza, John Miller, Theresa Reiker, Thomas P. Eisele, Thomas A Smith

# Additional File 1. Parameterisation of the models using Zambian field data

Each individual in the field survey for whom a positive RDT was reported, was used to define the household of residence of an index case in a simulated RCD program, Hypothetical index cases were sampled from the infections detected in the surveys. A set of concentric circles was defined around each index case, and for each radius *r*, the number of people residing within that radius, the total numbers tested in the survey, and the numbers positive for *P. falciparum*, were computed. The data were grouped into 10 categories defined by different values of *r*, and aggregated over all possible index cases. Corresponding to each of these radii, values of ν were computed as the mean, for all the index cases, of the number of individuals registered in the census within that radius (Table S1).

#### Table S1. Numbers of individuals registered by distance from index case

| Radius (km) | Mean* |
| --- | --- |
| 0.01 | 13.7 |
| 0.02 | 17.1 |
| 0.04 | 23.1 |
| 0.08 | 32.4 |
| 0.16 | 54.0 |
| 0.32 | 106.0 |
| 0.64 | 208.0 |
| 1.28 | 416.6 |
| 2.56 | 892.5 |
| 5.12 | 2002.2 |

The values refer to 1117 index cases including both surveys and all three trial arms.

For each index case, the corresponding prevalence for the largest value of $\nu$ provided an estimate of the local prevalence, *p*. The values of *p* thus obtained were binned into a series of categories, and the numbers of RDTs ($n_{i}$) and of positive RDTs ($r_{i})$were summed over all index cases for each range of *p* and of $\nu$. Estimates, $\hat{\tau}(\nu,p)$, of τ for each for each range of *p* and of $\nu$ were obtained as ratio of the local prevalence around any index case, to *p* i.e.:

$\hat{\tau}\left( \nu,p \right)=\left( \frac{\sum r_{i}}{{\sum n}_{i}} \right)/p$.

Equivalently, taking logarithms:

$$\log\left( \frac{\sum r_{i}}{{\sum n}_{i}} \right)=log\left( p \right)+ \log\left( \hat{\tau}(\nu,p) \right)$$

#### **Figure S1. Empirical Relationship of** $\hat{\boldsymbol{\tau}}\boldsymbol{(\nu,p)}$**with p and** $\boldsymbol{\nu}$**.**
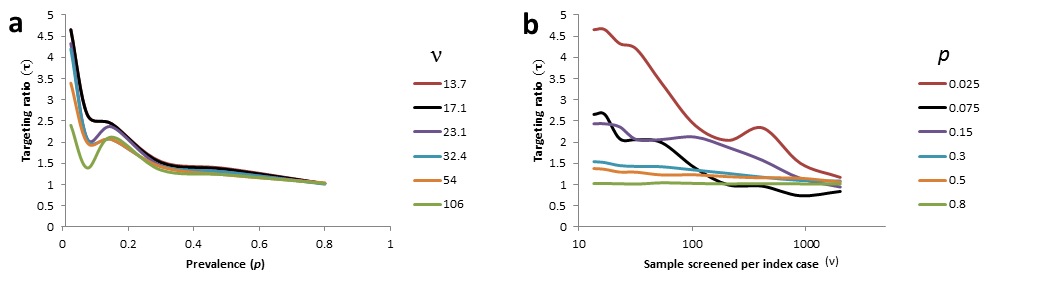


Empirically, $\hat{\tau}(\nu,p)$ was seen to decline steeply with *p* (Figure S1a) and also with $\nu$. The strong relationship with $\nu,$ applied only to low *p v*alues. At high prevalence even very focal screening is associated with a targeting ratio close to one.

For parameterisation of the models a smooth function of *p* and of $\nu$ was required as an estimator of $\tau\left( \nu,p \right)$. Several different functional relationships were fitted to the observed relationships between the numbers of infections found, and the numbers of individuals tested (using a Bayesian approach in WinBUGS(Spiegelhalter et al. 2003)). An excellent fit was observed with the function:

$$\tau\left( \nu,p \right)=\exp\left( \left( -\alpha_{1}\ln\left( p \right)+\frac{\alpha_{2}}{\nu}-\frac{\alpha_{3}}{\nu}\ln\left( p \right) \right)\frac{N-\nu}{N} \right) | \tau\left( \nu,p \right)\geq1$$

This function satisfies the important constraints that $\tau\left( N,p \right)=1,$since there can be no targeting if the whole population is tested. The estimates of the three parameters were: $\alpha_{1}$ = 0.23 (95% credible interval (CI): 0.16, 0.29); $\alpha_{2}$ = -1.40 (CI : -2.77, -0.02) and $\alpha_{3}$ = 2.87 (CI: 1.13, 4.59). Figure S2 is a heatmap, showing how $\tau\left( \nu,p \right)$ varies jointly with $\nu$ and $p$. Consistent with Figure S1, $\tau\left( \nu,p \right)$ is close to unity over most of the surface, but increases sharply when $\nu$ and $p$ are small.

##### **Figure S2. Fitted relationship of** $\boldsymbol{\tau}\left( \boldsymbol{\nu,}\boldsymbol{p} \right)$ **with** $\boldsymbol{p}$ **and** $\boldsymbol{\nu}$**.**


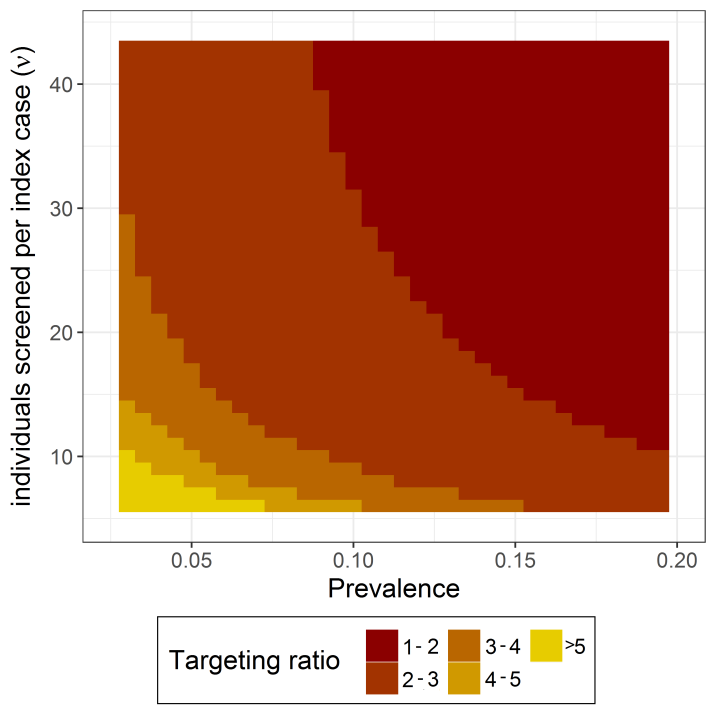


## References

Spiegelhalter, D. J., Thomas, A., Best, N., & Lunn, D. Winbugs Version 1.4. 2003. Cambridge, England, MRC-BSU.
